# Biologic treatment of Japanese patients with inflammatory bowel disease

**DOI:** 10.1186/s12876-018-0892-x

**Published:** 2018-11-01

**Authors:** Jörg Mahlich, Katsuyoshi Matsuoka, Rosarin Sruamsiri

**Affiliations:** 1 Health Economics & Outcomes Research, Janssen, Johnson & Johnson Platz 1, 41470 Neuss, Germany; 2 Düsseldorf Institute for Competition Economics (DICE), University of Düsseldorf, Düsseldorf, Germany; 3 Department of Gastroenterology and Hepatology, Toho University Sakura Medical Center, Sakura, Japan; 4 Center of Pharmaceutical Outcomes Research, Naresuan University, Phitsanulok, Thailand

**Keywords:** Japan, IBD, Biologic treatment

## Abstract

**Background:**

There is little information regarding the use of biologics in Inflammatory Bowel Disease (IBD) patients in Japan. The aim of this study was to determine the factors associated with the use of biologics in the treatment of Japanese patients with IBD.

**Methods:**

An online survey was conducted among Japanese patients with IBD (*n* = 1035). Socioeconomic as well as treatment related information was collected. Logistic regression was applied to analyze the determinants of biologic treatment.

**Results:**

Younger age (≤ 40 years vs. > 65 years; OR:0.24), time since diagnosis (< 2 years vs. < 15 years; OR: 4.16), surgical history (OR:1.98) and visiting university hospitals (university hospitals vs. clinics; OR: 0.47) were associated with biologic treatment for Japanese IBD patients.

**Conclusions:**

Currently, biologics have been used in younger IBD patients which may give rise to the presence of an age bias in biologic treatment. Further studies are required to confirm these results and to define appropriate IBD patients who should be treated with biologic agent.

## Background

Inflammatory bowel disease (IBD) is a lifelong condition that is considered to be one of the most prevalent inflammatory gastrointestinal diseases [1]. Although the precise cause of IBD remains unclear, it was suggested that dysregulated immune responses might play an important role in the development of IBD [2]. Crohn’s disease (CD) and ulcerative colitis (UC) are the two principal forms of IBD. In Japan, the prevalence of UC is 140,000 and that of CD 40,000 according to the Ministry of Health Labor and Welfare. However, those numbers do not include patients with mild to moderate IBD and therefore the actual number of IBD patients in Japan is estimated to be 20% to 40% higher [3]. IBD constitutes a significant burden to patients and it has been shown that Japanese patients with IBD face a four times higher risk of unemployment compared to the general population [4]. Treatment goals in IBD usually include sustained clinical remission, and a decrease in hospitalization and surgery. The pharmaceutical treatment of IBD includes anti-inflammatory drugs (5-aminosalicylic acid [5-ASA] such as mesalamine and mesalazine, and corticosteroids including prednisone and budesonide), immunomodulators (azathioprine, mercaptopurine, and methotrexate), antibiotics (metronidazole, tinidazole, ciprofloxacin, and clarithromycin), and biologic agents [5]. While steroids are effective for short-term treatment, they are less potent over the long-term and are associated with numerous side effects [6]. Whiole mesalazine is not effective in CD it is widely used for the maintenance of remission in mild UC [7]. Immunmodulators are an effective treatment option as their use is associated with 40% sustained remission over 1 year. On the other hand, they do not modify the natural disease course and are associated with low rates of endoscopic healing [8]. Therefore, biologics are a treatment option for patients with chronic active IBD that have incomplete mucosal healing under conventional treatment.

Currently (2016), five biologic agents have been approved by US FDA for the treatment of IBD (CD: infliximab, adalimumab, certolizumab pegol and ustekinumab and UC: infliximab, adalimumab and golimumab) [9], while only infliximab and adalimumab are approved for both UC and CD treatment in Japan. Biologics can induce sustained clinical remission, avoid the chronic use of steroids, reduce hospitalizations, and potentially prevent surgical intervention which often cause further complications [10, 11]. Initially, biologics were administered primarily to patients in a severe disease state [11]. In clinical trials of infliximab for CD, time since diagnosis was a mean of 8 years, the majority of patients already had surgery, and a third failed to respond to thiopurines [12]. There is a consensus that the most important factor for the optimization of therapy for IBD at treatment initiation with biologics is the ascertainment of active IBD inflammation [13].

Previous studies identified determinants of the prescription of biologic agents such as age, disease progression, socio-demographic variables, and the type of medical service provider. With regard to age, the elderly were reported to have lower biologic treatment rates in IBD compared to the younger generation [14, 15]. Although the response rate to biologic agents is similar in the elderly to that in younger patients [16], some studies suggested that the absolute risk of complications and side effects were higher in the elderly [17]. For socio-demographic variables, previous research has shown that even in developed countries with universal health coverage, access to the health care system is biased towards the wealthy [18]. In addition, some types of medical institutions such as university hospitals may gain experience of disease earlier and may therefore adapt to new medical technologies more rapidly than other medical institutions [19]. Furthermore, different providers face different incentives and have their own intrinsic views about treatment [20].

Currently, little is known about the use and the prescription determinants of biologics for the treatment of IBD in Japan. An enhanced understanding of the use of biologic agents has led to the optimized treatment of IBD patients. Against this background, this study assesses the determinants of treatment with biologic agents by using a survey of Japanese IBD patients all over Japan.

## Methods

### Study population

This study was a secondary data analysis of Morishige et al. [21] that was based on a nationwide online survey of 1035 patients. The survey was undertaken by IPSOS Healthcare, Inc., Tokyo, Japan, that runs a database containing 2,263,991 Japanese individuals. Inclusion criteria for this study were patients diagnosed with inflammatory bowel diseases who were currently receiving treatment. Patients working in pharmaceutical or medical companies, or marketing research/advertising agencies were excluded from participation. The patients were asked for their basic clinical characteristics (diagnosis, age, and gender), their socioeconomic status (marital status, income, work status, and educational level), their medical history (disease duration, surgical history, and comorbidity) and details on current treatment (type of treatment, frequency of physician’s office visits, and type of hospital).

### Statistical analysis

Descriptive statistics were tabulated using either the Chi-square or t-test to determine significant differences. To calculate factors associated with a treatment with biologics, we performed adjusted logistic regressions and reported the results as odds ratios (OR) with 95% confidence intervals (CI). An OR greater than one indicated that IBD patients with specific characteristic had a higher chance of either biologic initiation or treatment switching. The analysis was undertaken using STATA, College Station, USA. A *p* value of 0.05 (two-sided) was considered statistically significant. All data in this study were de-identified by the database provider.

## Results

### Study population and medication

Table 1 shows the descriptive statistics of the included patients. The majority of the patients (77%) were diagnosed with UC while 23% had CD. In addition, 17% of IBD patients currently use biologic agents. The majority of patients were male between 41 and 65 of age.

**Table 1:** Characteristics of IBD patients with or without biologic treatment

Characteristics	Crohn’s disease				Ulcerative Colitis			
	All, n (%)	Biologics users, n (%)	Non-biologics users, n (%)	*P*-value	All, n (%)	Biologics users, n (%)	Non-biologics users, n (%)	P-value
Patients	235 (23)	113 (48)	122 (52)		800 (77)	63 (8)	737 (92)	
Age (mean ± SD)	42.27 ± 9.95	40.82 ± 9.09	44.00 ± 10.49	0.014	46.19 ± 10.83	45.25 ± 11.83	46.27 ± 10.74	0.474
≤ 40 years	102 (43)	55 (49)	47 (39)		251 (31)	24 (38)	227 (31)	
41–65 years	124 (53)	55 (49)	69 (57)		466 (58)	34 (54)	432 (59)	
> 65 years	9 (4)	3 (2)	6 (4)		83 (11)	5 (8)	78 (10)	
Gender				0.521				0.285
Male	168 (71)	83 (73)	85 (70)		507 (63)	36 (57)	471 (64)	
Female	67 (29)	30 (27)	37 (30)		293 (37)	27 (43)	266 (36)	
Marriage status				0.253				0.335
Married	124 (53)	64 (57)	60 (49)		261 (33)	24 (38)	237 (32)	
Single	111 (47)	49 (43)	62 (51)		539 (77)	39 (62)	500 (68)	
Highest Education				0.010				0.973
College or less	136 (58)	73 (65)	63 (52)		383 (48)	31 (49)	352 (48)	
Bachelor’s degree	84 (36)	38 (34)	46 (38)		363 (45)	28 (44)	335 (45)	
Master’s degree	15 (6)	2 (1)	13 (10)		54 (7)	4 (6)	50 (7)	
Occupation				0.645				0.986
Fulltime	130 (55)	64 (57)	66 (54)		450 (56)	34 (54)	416 (56)	
Part-time	19 (8)	8 (7)	11 (9)		76 (10)	7 (11)	69 (9)	
Self employed	20 (9)	7 (6)	13 (11)		59 (7)	6 (10)	53 (7)	
House wife/house keeper	24 (10)	11 (10)	13 (11)		103 (13)	8 (13)	95 (13)	
Student	2 (1)	2 (2)	0 (0)		2 (0)	0 (0)	3 (0)	
Unemployed/Pensioner	38 (16)	20 (18)	18 (15)		94 (12)	7 (11)	87 (12)	
Others	2 (1)	1 (0)	1 (0)		15 (2)	1 (1)	14 (3)	
Household incomes				0.967				0.427
< 2 M JPY	23 (10)	11 (10)	12 (10)		72 (9)	4 (6)	68 (9)	
2–4 M JPY	47 (20)	21 (19)	26 (21)		144 (18)	13 (21)	131 (18)	
4–6 M JPY	55 (24)	25 (22)	30 (25)		185 (23)	16 (26)	169 (23)	
6–8 M JPY	36 (15)	20 (18)	16 (13)		135 (17)	9 (14)	126 (17)	
8–10 M JPY	24 (10)	11 (10)	13 (11)		75 (9)	2 (3)	73 (10)	
> 10 M JPY	19 (8)	9 (8)	10 (8)		85 (11)	7 (11)	78 (11)	
Not revealed	31 (13)	16 (13)	15 (12)		104 (13)	12 (19)	92 (12)	
Comorbidity								
Dyslipidemia	34 (14)	9 (8)	25 (20)	0.006	83 (10)	10 (16)	73 (10)	0.136
Hypertension	38 (16)	10 (9)	28 (23)	0.003	108 (14)	15 (24)	93 (13)	0.013
COPD Asthma	13 (6)	5 (4)	8 (7)	0.475	28 (4)	9 (14)	19 (3)	< 0.001
Diabetes	16 (7)	5 (4)	11 (9)	0.163	56 (7)	13 (21)	43 (6)	< 0.001
Depression	17 (7)	5 (4)	12 (10)	0.110	48 (6)	4 (6)	44 (6)	0.903
OA and RA	12 (5)	4 (4)	8 (7)	0.294	32 (4)	9 (14)	23 (3)	< 0.001
Gastrointestinal disease	15 (6)	5 (4)	10 (8)	0.237	49 (6)	11 (18)	38 (5)	< 0.001
Current treatment								
Nutritional	137 (58)	65 (58)	72 (59)	0.816	107 (13)	18 (28)	89 (12)	< 0.001
5-ASA	168 (71)	82 (73)	86 (70)	0.725	656 (82)	56 (89)	600 (81)	0.138
Steroid	39 (17)	16 (14)	23 (19)	0.334	127 (16)	21 (33)	106 (14)	< 0.001
immunomodulator	53 (23)	30 (27)	23 (19)	0.158	61 (8)	27 (43)	64 (9)	< 0.001
Biologic agents	112 (48)	–	–		56 (7)	–	–	
Cytapheresis	1 (0)	0 (0)	1 (1)	0.335	16 (2)	7 (11)	9 (1)	< 0.001
Surgical history								
Yes	146 (62)	72 (64)	74 (61)	0.629	94 (12)	13 (21)	81 (11)	0.023
Time since diagnosis				0.179				0.267
0–2 years	27 (11)	8 (7)	19 (16)		134 (17)	7 (11)	127 (17)	
3–8 years	66 (28)	34 (30)	31 (25)		296 (37)	30 (48)	266 (36)	
9–15 years	44 (19)	24 (21)	20 (16)		197 (25)	15 (24)	182 (25)	
> 15 years	99 (42)	47 (42)	52 (43)		173 (22)	11 (17)	162 (22)	
Type of hospitals				0.657				0.001
University hospital	92 (39)	41 (36)	51 (42)		160 (20)	25 (40)	135 (18)	
General/Specialist hospital	128 (55)	65 (57)	63 (52)		410 (51)	26 (41)	384 (52)	
Clinics	15 (6)	7 (7)	8 (6)		229 (29)	12 (19)	217 (30)	
Others	0 (0)	0 (0)	0 (0)		1 (0)	0 (0)	1 (0)	
Frequency of visit				< 0.001				< 0.001
More than once a month	38 (16)	11 (10)	27 (22)		61 (8)	16 (24)	46 (6)	
Once every month	73 (31)	29 (26)	44 (36)		281 (35)	27 (43)	254 (35)	
Once every 2 months	94 (40)	69 (61)	25 (20)		247 (31)	20 (32)	227 (31)	
Less than every 2 months	30 (13)	4 (3)	26 (22)		211 (26)	1 (1)	210 (28)	

COPD, chronic obstructive pulmonary disease; OA, osteoarthritis; RA, rheumatoid arthritis; M JPY, million Japanese Yen; SD, standard deviation

### Determinants of biologic treatment

Table 2 shows the results of the logistic regression. The coefficients are expressed as OR and bold numbers indicate significance at the 5% level.

**Table 2:** Determinants of biologic treatment and regression results

Characteristics	Overall, ORs, (95%CI)	Crohn’s disease, ORs (95%CI)	Ulcer Colitis, ORs (95%CI)
Age (reference ≤40 years)			
41–65 years	**0.59 (0.37–0.93)**	**0.41 (0.17–0.95)**	0.86 (0.42–1.74)
> 65 years	**0.24 (0.09–0.65)**	0.37 (0.05–2.85)	0.31 (0.08–1.26)
Gender (reference male)			
Female	0.77 (0.46–1.29)	**0.30 (0.11–0.79)**	1.67 (0.77–3.63)
Marriage status (reference married)			
Single	0.68 (0.41–1.10)	0.87 (0.37–2.04)	1.29 (0.60–2.81)
Highest Education (reference college or less)			
Bachelor’s degree	0.76 (0.49–1.18)	0.51 (0.22–1.19)	1.01 (0.51–2.02)
Master’s degree and higher	**0.26 (0.10–0.71)**	**0.06 (0.01–0.41)**	1.45 (0.41–5.16)
Occupation			
Fulltime	2.74 (0.43–7.13)	0.38 (0.01–1.17)	2.38 (0.25–3.00)
Part-time	2.16 (0.32–4.70)	0.28 (0.01–1.07)	1.57 (0.14–17.08)
Self employed	2.39 (0.34–6.52)	0.21 (0.01–7.28)	3.21 (0.28–6.28)
House wife	3.24 (0.46–2.52)	0.25 (0.01–1.69)	1.95 (0.17–2.24)
Student	5.20 (0.30–9.37)	0 (Omitted)	0 (Omitted)
Unemployed/Pensioner	3.50 (0.54–2.65)	0.77 (0.02–5.11)	2.44 (0.22–6.58)
Household incomes (reference less than 2 M JPY)			
2–4 M JPY	1.26 (0.55–2.87)	0.25 (0.06–1.03)	1.49 (0.40–5.59)
4–6 M JPY	1.56 (0.69–3.56)	1.04 (0.28–3.86)	1.16 (0.29–4.56)
6–8 M JPY	1.50 (0.62–3.61)	1.98 (0.45–8.83)	0.74 (0.17–3.22)
8–10 M JPY	1.43 (0.51–4.01)	0.84 (0.17–4.27)	0.57 (0.08–4.09)
> 10 M JPY	1.24 (0.45–3.41)	1.05 (0.18–6.02)	0.51 (0.09–2.71)
Comorbidity			
Dyslipidemia	0.55 (0.23–1.34)	0.73 (0.13–3.96)	0.36 (0.09–1.56)
Hypertension	0.85 (0.42–1.72)	0.41 (0.11–1.60)	1.20 (0.44–3.28)
COPD Asthma	1.70 (0.62–4.61)	3.32 (0.39–8.54)	2.00 (0.53–7.57)
Diabetes	1.42 (0.60–3.33)	0.97 (0.15–6.34)	2.48 (0.80–7.64)
Depression	0.64 (0.27–1.49)	0.49 (0.10–2.30)	0.61 (0.16–2.36)
OA and RA	1.27 (0.29–5.52)	0.41 (0.04–4.45)	2.44 (0.24–24.85)
Gastrointestinal disease	1.03 (0.41–2.57)	0.69 (0.10–4.55)	1.39 (0.39–4.93)
Surgical history			
Previous surgical history	**1.98 (1.24–3.16)**	1.15 (0.53–2.47)	0.82 (0.28–2.36)
Current treatment			
Nutritional	**3.16 (2.00–5.12)**	1.90 (0.87–4.12)	2.42 (1.00–5.89)
5-ASA	0.89 (0.54–1.49)	1.05 (0.46–2.39)	1.24 (0.46–3.35)
Steroid	1.04 (0.61–1.78)	2.25 (0.81–6.26)	1.19 (0.56–2.56)
Immunosuppressant	**3.15 (1.94–5.12)**	1.44 (0.62–3.28)	**4.61 (2.25–9.45)**
Cytapheresis	1.23 (0.33–4.64)	0 (Omitted)	2.00 (0.41–9.68)
Time since diagnosis (reference 0–2 years)			
3–8 years	**3.63 (1.69–7.76)**	1.48 (0.33–6.54)	**4.27 (1.33–3.72)**
9–15 years	**3.26 (1.42–7.45)**	1.10 (0.22–5.52)	**3.99 (1.13–4.13)**
> 15 years	**4.16 (1.80–9.56)**	0.92 (0.20–4.28)	**3.95 (1.04–4.90)**
Type of hospitals (reference university hospital)			
General/Specialist hospital	**0.62 (0.40–0.96)**	1.09 (0.50–2.34)	**0.41 (0.19–0.84)**
Clinics	**0.47 (0.24–0.89)**	0.42 (0.10–1.79)	0.43 (0.17–1.09)
Frequency of visit (Reference less than once in a month)			
Once every month	0.91 (0.45–1.84)	1.56 (0.47–5.22)	0.58 (0.22–1.56)
Once every 2 months	1.83 (0.88–3.82)	**14.36 (3.98–51.83)**	0.50 (0.17–1.45)
Once every 3 months	0.09 (0.03–0.27)	0.26 (0.05–1.21)	**0.03 (0.01–0.29)**
Less than every 3 months	0 (Omitted)	0 (Omitted)	0 (Omitted)

*COPD* chronic obstructive pulmonary disease, *OA* osteoarthritis, *RA* rheumatoid arthritis, *M JPY* million Japanese Yen, *OR* odds ratio, *CI* confidence interval

Bold numbers indicate significance at 5% level

Elderly patients had lower chance of receiving biologic agents compared to IBD patients aged ≤40 years (IBD patients aged > 65 years, OR 0.24 (95% CI; 0.09–0.64). Patients with a diagnosis within 2 years have a higher probability of recieving biologic agents compared to those with more longstanding disease. (patients diagnosed 3–8 years, OR 3.63 (95% CI 1.69–7.76) and patients diagnosed > 15 years, OR 4.16 (95% CI 1.80–9.56). IBD patients who visited a university hospital and had previous surgical experience had a higher chance of receiving biologic treatment. In Crohn’s disease, male IBD patients have higher probability of using biologic agents than women. Marriage status, occupation, household income, comorbidity, and frequency of hospital visit did not play role in the use of biologic agents.

## Discussion

To our knowledge, this is the first study to determine the factors associated with the use of biologics for the treatment of Japanese patients with IBD. Younger IBD patients, a long time since diagnosis, surgical history, and visiting university hospitals were the key determinant factors for receiving biologic agents for Japanese IBD patients. Previous research in Japan also demonstrated that IBD patients who are treated with a biologics are also very fond of being involved in the treatment decision [21] Involvement in the treatment decision in turn corresponds to higher levels of patient’s treatment satisfaction [22].

Both sex and age distribution of our sample resembles the overall Japanese IBD patient population with highest prevalence in the age cohort patients being in their 30’s and 40’s [23].

We confirmed that younger age is significantly associated with biologic treatment. In other indications, these findings might also be interpreted as evidence of the so-called “age bias”, which refers to the observation that older patients are less likely to get access to innovative medical treatment compared to younger patients with the same disease severity. Indeed, some studies outside Japan found evidence for the presence of an age bias in rheumatoid arthritis [24–26]. For Japan, age was also found to be negatively correlated with the initiation of biologic treatment in RA [27].

A possible explanation for this is the higher sensitivity of older patients to the immunosuppressive side effects of currently available biologics. Because biologic drugs suppress the immune system, infection is one of the most common side effects [28]. Indeed, some studies have suggested a positive relationship between age and the risk of side effects in IBD [29, 30]. Accordingly, the elderly are more likely to discontinue treatment [31]. Based on these previous findings, Japanese physicians might be more risk averse with regard to using more aggressive treatment options for older patients. Risk aversion had been reported as a key characteristic of the Japanese culture in general [32] which has also a significant impact on the health care sector [33]. However, alternative treatments such as corticosteroids and urgent surgery are also associated with a higher absolute risk for complications in the elderly [34]. Therefore, it is not clear to which degree the reduced use of biologics in the elderly population might decrease the overall risk of complications.

For the CD subpopulation we found female gender to be related to biologic treatment which might reflect the better response rates of biologics in females [35].

Annual income did not play a role in selecting biologic treatment in Japan which was different from previous findings in other developed countries [36]. The main reason that can explain this finding is that IBD is classified as an intractable disease (“nanbyo”) by the Japanese government. For those diseases, the government provides partial financial assistance and patients will pay only 20% of their medical expenses up to a maximum of between JPY 3000 and JPY 44,000/month depending on income. For diseases that are not on the intractable disease list, co-payment ceilings vary between JYP 35,400 and JYP 252,600. Hence the IBD patient can use biologic agents for lower cost when compare to other indications such as RA [37].

We also found that patients with previous surgery were more likely to receive biologics, possibly because this is associated with a lower recurrence of CD in patients treated postoperatively with anti-TNF-α antibodies [38, 39]. In addition, receiving biologic treatment after post-surgery, this can also help to prevent re-surgery especially in patients with more aggressive disease and high risk of recurrence [40]. Surgical history is one of the few variables where results differ between CD and UC patients. Probably, having a surgical history in UC means that the patient is cured while surgical history in CD can be an indicator of the disease complexity.

Time since diagnosis is another major determinant of biologic treatment. This finding is also in line with the idea that biologics are primarily used in later treatment lines after the failure of previous treatments [41]. This is mainly true for UC patients, which reflects the popularity of the traditional step up treatment approach [42]. In CD patients, there was no clear relationship between time since diagnosis and biologic use, because disease progression is not linear [43].

From a medical point of view, it has been suggested that an early treatment initiation with biologic agents would be beneficial for patients because the overall remission rates with biologics are numerically greater when administered to patients within 2 years of diagnosis [44]. Therefore, it is argued that the preferred candidate for biologic treatment is a patient with confirmed active intestinal inflammation prior to the development of complications such as strictures [11]. Our results appear to be inconsistent with this recommendation.

Steroid use has been identified as a factor that increases the relative risk of complications with biologics in CD [45]. Therefore, we expected that the use of steroids would be associated with a reduced use of biologics. However, contrary to our expectations, this was not been confirmed. The most likely explanation of that finding is that severe patients receive both steroids and biologics at the same time. Our results suggest that the only two co-medications that increased the likelihood of being treated with a biologic are the use of immunomodulators and nutritional treatment in the overall population.

Finally, we found that the health care provider played a significant role in the treatment decision. Patients who were treated in university hospitals had the highest chance of being treated with a biologic, but those treated in general clinics had the lowest chance. Acknowledging the potential endogeneity of this variable, we interpret this finding as support for the hypothesis that university hospitals are more likely to adapt new medical technologies. Moreover, as physicians in tertiary institutions see more patients with a special disease, they might develop confidence and treatment protocols that lead to more pro-active treatment with the available technologies.

Several limitations should be noted. First, this online study was conducted based on a cross sectional survey performed on a random sample of IBD patients all over Japan, and people that are more familiar with the Internet have a higher probability of taking part such surveys. Therefore, respondents might be better educated than the general IBD population. Second, we collected patient-reported data. Therefore, a limited set of medical parameters such as severity can be collected. However, some factors such as time since diagnosis and surgical history can be used as predictors of IBD severity. Last, we could not determine the time of starting a biologic agent from the survey. Therefore, we could not determine the temporal relationship between factors and biologic use.

To optimize IBD treatment, further studies involving a large cohort of patients are required to confirm these data and to define precise determinants for assessing and managing treatment in IBD patients treated with biologic agents. Those studies would need a methodological framework that allows for a more robust investigation of treatment choices and would also need to include more medical parameters such as a more precise measure of disease severity, time since initial biologic treatment, and the reason for stopping biologic treatment, to ensure causality. For instance, Siegel et al. suggested a set of attributes such as presence of mucosal lesions in CD to come up with a novel overall disease severity measure that could be built on in future research [46].

## Conclusion

Key determinant factors for receiving biologic agents for Japanese IBD patients are younger IBD patients, time since diagnosis, male gender (CD only), surgical history, and visiting a university hospital. Further studies are required to confirm these data and to define precise determinants for assessing and managing treatment in IBD patients. The findings of this study will help physicians identify suitable IBD patients who require biologic agents and improve patient treatment outcomes in the future.

## References

[1] Korzenik JR, Podolsky DK (2006). Evolving knowledge and therapy of inflammatory bowel disease. Nat Rev Drug Discov.

[2] Xavier RJ, Podolsky DK (2007). Unravelling the pathogenesis of inflammatory bowel disease. Nature.

[3] Kanai T, Matsuoka K, Naganuma M, Hayashi A, Hisamatsu T (2014). Diet, microbiota, and inflammatory bowel disease: lessons from Japanese foods. Korean J Intern Med.

[4] Mahlich J, Matsuoka K, Nakamura Y, Sruamsiri R (2017). The relationship between socio-demographic factors, health status, treatment type, and employment outcome in patients with inflammatory bowel disease in Japan. BMC Public Health.

[5] Triantafillidis J, Merikas E, Georgopoulos F (2011). Current and emerging drugs for the treatment of inflammatory bowel disease. Drug Des Devel Ther.

[6] D'Haens G, Baert F, van Assche G, Caenepeel P, Vergauwe P, Tuynman H, De Vos M, van Deventer S, Stitt L, Donner A (2008). Early combined immunosuppression or conventional management in patients with newly diagnosed Crohn's disease: an open randomised trial. Lancet.

[7] Kornbluth A, Sachar DB (2004). Practice parameters Committee of the American College of gastroenterology: ulcerative colitis practice guidelines in adults (update): American College of Gastroenterology, Practice Parameters Committee. Am J Gastroenterol.

[8] Cosnes J, Nion-Larmurier I, Beaugerie L, Afchain P, Tiret E, Gendre JP (2005). Impact of the increasing use of immunosuppressants in Crohn's disease on the need for intestinal surgery. Gut.

[9] Sandborn W (2016). The present and future of inflammatory bowel disease treatment. Gastroenterol Hepatol (N Y).

[10] Mandel MD, Miheller P, Müllner K, Golovics PA, Lakatos PL (2014). Have biologics changed the natural history of Crohn's disease?. Dig Dis.

[11] Moss AC (2015). Optimizing the use of biological therapy in patients with inflammatory bowel disease. Gastroenterol Rep (Oxf).

[12] Hanauer SB, Feagan BG, Lichtenstein GR, Mayer LF, Schreiber S, Colombel JF, Rachmilewitz D, Wolf DC, Olson A, Bao W (2002). Maintenance infliximab for Crohn's disease: the ACCENT I randomised trial. Lancet.

[13] Ben-Horin S, Mao R, Chen M (2015). Optimizing biologic treatment in IBD: objective measures, but when, how and how often?. BMC Gastroenterol.

[14] Bautista MC, Otterson MF, Zadvornova Y, Naik AS, Stein DJ, Venu N, Perera LP (2013). Surgical outcomes in the elderly with inflammatory bowel disease are similar to those in the younger population. Dig Dis Sci.

[15] Lakatos P, David G, Pandur T (2011). IBD in the elderly population: results from a population-based study in Western Hungary, 1977-2008. J Crohn's Colitis.

[16] Moleski SM, Lindenmeyer CC, Kozuch PL. The use of infliximab in older inflammatory bowel disease patients. Gastroenterology. 2011;S-361.

[17] Ljung T (2004). Infliximab in inflammatory bowel disease: clinical outcome in a population based cohort from Stockholm County. Gut.

[18] Van Doorslaer E, Masseria C, Koolman X (2006). Inequalities in access to medical care by income in developed countries. CMAJ.

[19] Calara P, Norlin J, Althin R, Steen Carlsson K, Schmitt-Egenolf M (2016). Healthcare provider type and switch to biologics in psoriasis: evidence from real-world practice. BioDrugs.

[20] Chandra ACD, Song ZH (2012). Who ordered that? The economics of treatment choices in medical care. Handb Health Econ.

[21] Morishige R, Nakajima H, Yoshizawa K, Mahlich J, Sruamsiri R (2017). Preferences regarding shared decision-making in Japanese inflammatory bowel disease patients. Adv Ther.

[22] Mahlich J, Matsuoka S, Sruamsiri R (2017). Shared decision making and treatment satisfaction in Japanese patients with inflammatory bowel disease (IBD). Dig Dis.

[23] Asakura K, Nishiwaki Y, Inoue N, Hibi T, Watanabe M, Takebayashi T (2009). Prevalence of ulcerative colitis and Crohn’s disease in Japan. J Gastroenterol.

[24] Fraenkel L, Rabidou N, Dhar D (2006). Are rheumatologists’ treatment decisions influenced by patients’ age?. Rheumatology.

[25] Radovits BJ, Fransen J, Eijsbouts A, Riel van P, Laan RF (2009). Missed opportunities in the treatment of elderly patients with rheumatoid arthritis. Rheumatology (Oxford).

[26] Kievit W, van Hulst L, van Riel P, Fraenkel L (2010). Factors that influence rheumatologists' decisions to escalate care in rheumatoid arthritis: results from a choice-based conjoint analysis. Arthritis Care Res (Hoboken).

[27] Mahlich J, Sruamsiri R (2017). Treatment patterns of rheumatoid arthritis in Japanese hospitals and predictors of the initiation of biologic agents. Curr Med Res Opin.

[28] Bonovas S, Fiorino G, Allocca M, Lytras T, Nikolopoulos GK, Peyrin-Biroulet L, Danese S (2016). Biologic therapies and risk of infection and malignancy in patients with inflammatory bowel disease: a systematic review and network meta-analysis. Clin Gastroenterol Hepatol.

[29] Cottone M, Kohn A, Daperno M, Armuzzi A, Guidi L, D'Inca R, Bossa F, Angelucci E, Biancone L, Gionchetti P (2011). Advanced age is an independent risk factor for severe infections and mortality in patients given anti-tumor necrosis factor therapy for inflammatory bowel disease. Clin Gastroenterol Hepatol.

[30] Bhushan A , Pardi DS , Loft us EV et al: Association of age with adverse events from biologic therapy in patients with infl ammatory bowel disease *Gastroenterology* 2010, **138**(Supplement):Abstract 413.

[31] Desai A, Zator Z, de Silva P, Nguyen D, Korzenik J, Yajnik V, Ananthakrishnan A (2013). Older age is associated with higher rate of discontinuation of anti-TNF therapy in patients with inflammatory bowel disease. Inflamm Bowel Dis.

[32] Yeh R (1989). On Hofstede's treatment of Chinese and Japanese values. Asia Pacific Journal of Business.

[33] Mahlich J, Dilokthornsakul P, Sruamsiri R, Chaiyakunapruk N (2018). Cultural beliefs, utility values, and health technology assessment. Cost Effectiveness and Resource Allocation.

[34] Ikeuchi H, Uchino M, Matsuoka H, Bando T, Hirata A, Takesue Y, Tomita N, Matsumoto T (2014). Prognosis following emergency surgery for ulcerative colitis in elderly patients. Surg Today.

[35] Billioud V, Sandborn WJ, Peyrin-Biroulet L (2011). Loss of response and need for adalimumab dose intensification in Crohn's disease: a systematic review. Am J Gastroenterol.

[36] Dewitt ES, Lin L, Glick HA, Anstrom KJ, Schulman KA, Reed SD (2012). Pattern and predictors of the initiation of biologic agents for the treatment of rheumatoid arthritis in the United States: an analysis using a large observational data Bank. Clin Ther.

[37] Ministry of Heath, Labour and Welfare. 難病 (nanbyou), Tokyo, 2017, available at: http://www.mhlw.go.jp/stf/seisakunitsuite/bunya/kenkou_iryou/kenkou/nanbyou

[38] Fernandez-Blanco I, Monturiol J, Martinez B e (2010). Adalimumab in the prevention of postoperative recurrence of Crohn’s disease. Gastroenterology.

[39] Regueiro M, Schraut W, Baidoo L, Kip KE, Sepulveda AR, Pesci M, Harrison J, Plevy SE (2009). Infliximab prevents Crohn's disease recurrence after ileal resection. Gastroenterology.

[40] Clarke K, Regueiro M (2009). Prevention and treatment options for postoperative Crohn’s disease: a clinical dilemma. Gastroenterol Hepatol (N Y).

[41] Tajima A (2014). Biologics for the treatment of inflammatory bowel disease (IBD). JSM Gastroenterol Hepatol.

[42] Sandborn W (2007). Step-up versus top-down therapy in the treatment of ulcerative colitis. Gastroenterol Hepatol (N Y).

[43] Freeman H (2014). Natural history and long-term clinical course of Crohn’s disease. World J Gastroenterol.

[44] Cornillie F, Hanauer S, Diamond RH (2014). Can clinical, biological or pharmacological markers predict sustained response to infliximab? A retrospective analysis of ACCENT 1. Gut.

[45] Beaugerie L, Sokol H (2012). Clinical, serological and genetic predictors of inflammatory bowel disease course. World J Gastroenterol.

[46] Siegel C, Whitman CB, Spiegel BMR, Feagan BG, Sand B, Loftus EV, Panaccione R, D'Haens G, Bernstein C, Gearry R (2018). Development of an index to define overall disease severity in IBD. Gut.

